# The genome sequence of the Brown China-mark moth, *Elophila nymphaeata *(Linnaeus, 1758)

**DOI:** 10.12688/wellcomeopenres.21118.1

**Published:** 2024-03-19

**Authors:** Douglas Boyes, Gavin R. Broad, Laura Sivess, Stephanie Holt, Peter W.H. Holland, University of Oxford and Wytham Woods Genome Acquisition Lab, Natural History Museum Genome Acquisition Lab, Darwin Tree of Life Barcoding collective, Wellcome Sanger Institute Tree of Life Management, Samples and Laboratory team, Wellcome Sanger Institute Scientific Operations: Sequencing Operations, Wellcome Sanger Institute Tree of Life Core Informatics team, Tree of Life Core Informatics collective, Darwin Tree of Life Consortium

**Affiliations:** 1 UK Centre for Ecology & Hydrology, Wallingford, England, UK; 2 Natural History Museum, London, England, UK; 3 University of Oxford, Oxford, England, UK

**Keywords:** Elophila nymphaeata, Brown China-mark moth, genome sequence, chromosomal, Lepidoptera

## Abstract

We present a genome assembly from an individual female *Elophila nymphaeata* (the Brown China-mark moth; Arthropoda; Insecta; Lepidoptera; Crambidae). The genome sequence is 734.1 megabases in span. Most of the assembly is scaffolded into 31 chromosomal pseudomolecules, including the Z and W sex chromosomes. The mitochondrial genome has also been assembled and is 15.3 kilobases in length. Gene annotation of this assembly on Ensembl identified 12,079 protein coding genes.

## Species taxonomy

Eukaryota; Opisthokonta; Metazoa; Eumetazoa; Bilateria; Protostomia; Ecdysozoa; Panarthropoda; Arthropoda; Mandibulata; Pancrustacea; Hexapoda; Insecta; Dicondylia; Pterygota; Neoptera; Endopterygota; Amphiesmenoptera; Lepidoptera; Glossata; Neolepidoptera; Heteroneura; Ditrysia; Obtectomera; Pyraloidea; Crambidae; Nymphulinae; *Elophila*; *Elophila nymphaeata* Linnaeus, 1758 (NCBI:txid753177).

## Background

The vast majority of Lepidoptera (moths and butterflies) have terrestrial larvae. There are several species, however, that lay eggs underwater with the larvae remaining aquatic and feeding on submerged plants. This trait is found in a few distinct lineages of Lepidoptera nested within the broad phylogenetic sweep of terrestrial species ( Pabis, 2018); it seems certain, therefore, that the aquatic lifestyle has evolved secondarily. The subfamily Acentropinae, family Crambidae, contains the majority of aquatic Lepidoptera including several species commonly called ‘china-mark moths’ due to their delicately patterned, shiny white wings likened to pottery.

The Brown China-mark moth *Elophila nymphaeata* is a widespread aquatic species found in wetland areas of Europe and across the Palaearctic to the far east of Russia ( GBIF Secretariat, 2024). In Britain and Ireland, the species is found in lowland regions close to static and slow-moving water such as ponds, lakes, bogs and canals ( National Biodiversity Data Centre, 2024; NBN Atlas Partnership, 2024). Eggs are laid on the underside of the leaves of aquatic plants. The larvae initially feed in leaf mines before constructing mobile cases formed from two oval-shaped leaf fragments cut from aquatic plants, spun together with silk ( Bierne, 1952; Meyrick, 1895; Rowland, 2018; Vallenduuk & Cuppen, 2004). The larvae feed on a range of aquatic plants, including *Hydrocharis* and *Potamogeton*, and in Italy the species has been recorded as a pest of rice plants ( Ugolini, 1991). There is generally one generation per year, with larvae feeding through autumn, overwintering and feeding again in spring; after a brief pupation, adults are seen in June and July.

A genome sequence of the Brown China-mark moth *Elophila nymphaeata* was determined as part of the Darwin Tree of Life project. The genome sequence will facilitate research into adaptations to aquatic life in insects and will contribute to the growing set of resources for studying molecular evolution in the Lepidoptera.

## Genome sequence report

**Figure 1.**
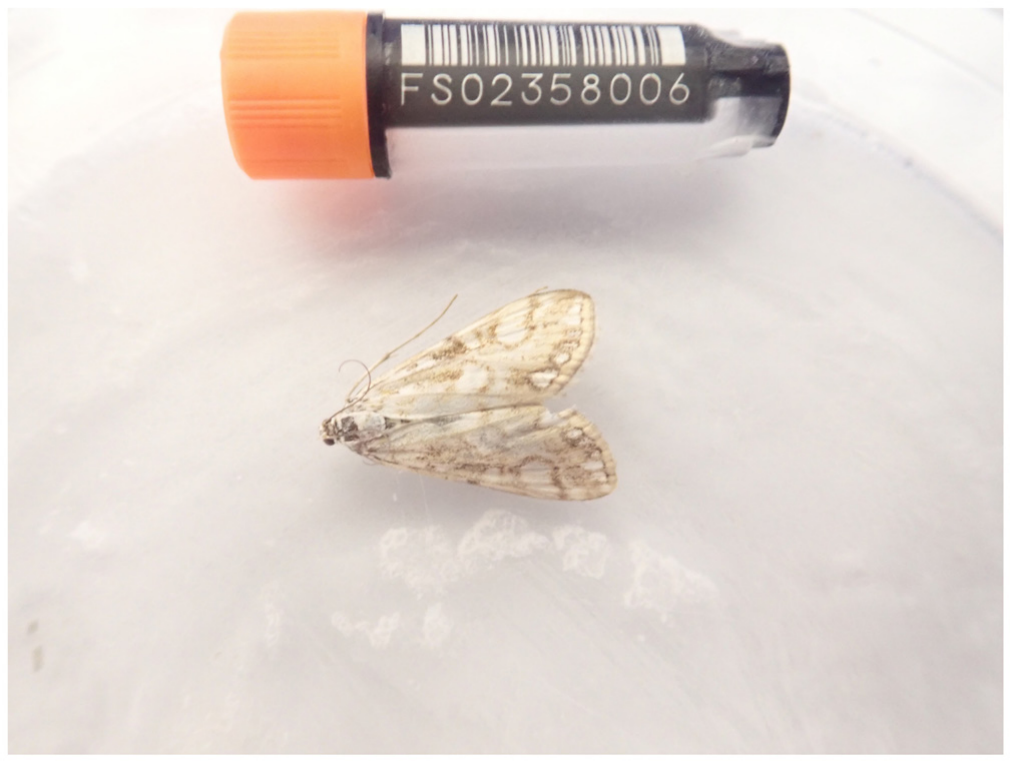
Photograph of the *Elophila nymphaeata* (ilEloNymp1) specimen used for genome sequencing.

The genome was sequenced from one female *Elophila nymphaeata* ( Figure 1) collected from Wytham Woods, Oxfordshire, UK (51.76, –1.34). A total of 66-fold coverage in Pacific Biosciences single-molecule HiFi long reads was generated. Primary assembly contigs were scaffolded with chromosome conformation Hi-C data. Manual assembly curation corrected 102 missing joins or mis-joins and removed 28 haplotypic duplications, reducing the assembly length by 2.96% and the scaffold number by 15.15%, also decreasing the scaffold N50 by 1.16%.

**Figure 2.**
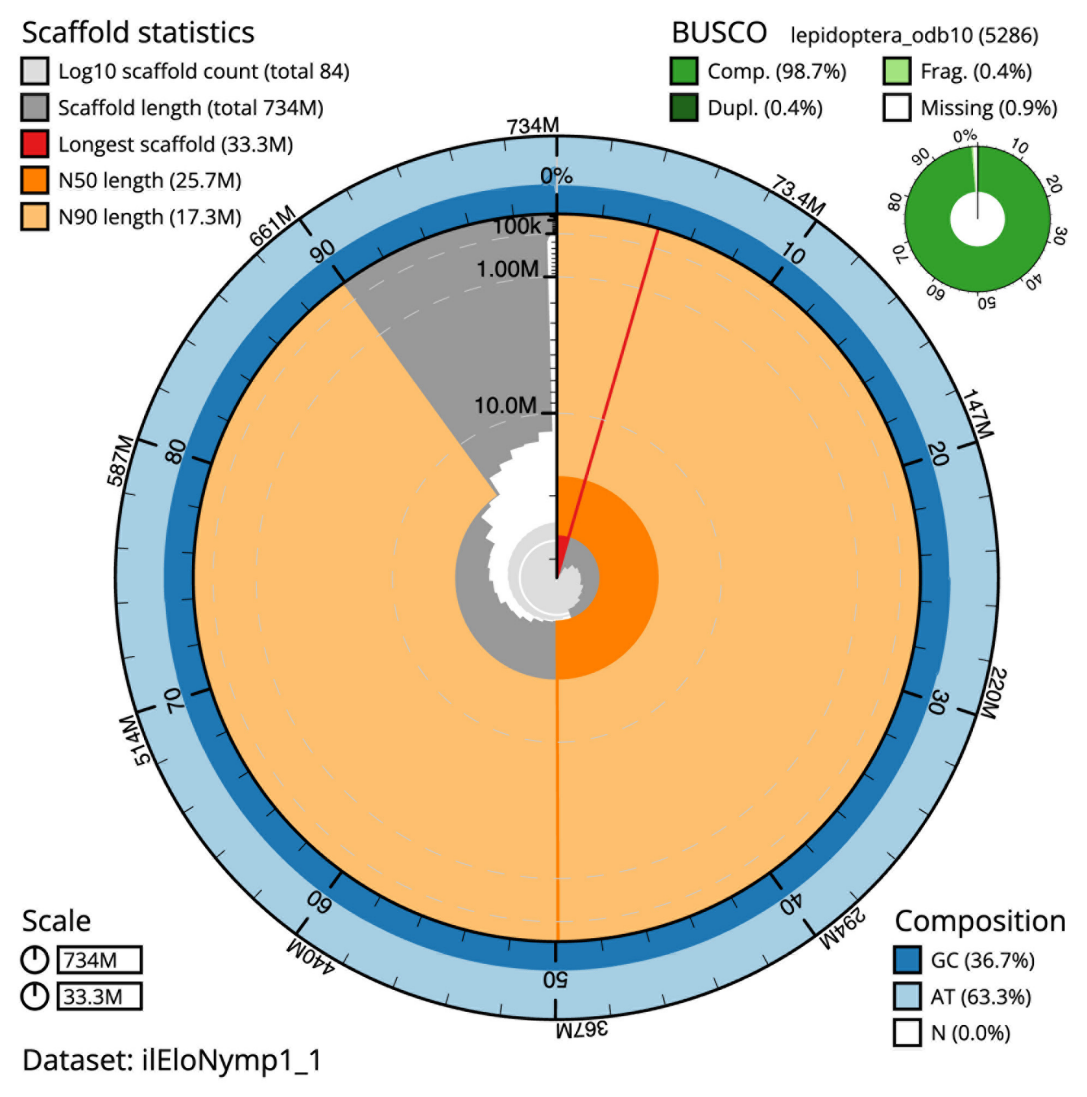
Genome assembly of *Elophila nymphaeata*, ilEloNymp1.1: metrics. The BlobToolKit snail plot shows N50 metrics and BUSCO gene completeness. The main plot is divided into 1,000 size-ordered bins around the circumference with each bin representing 0.1% of the 734,146,407 bp assembly. The distribution of scaffold lengths is shown in dark grey with the plot radius scaled to the longest scaffold present in the assembly (33,319,495 bp, shown in red). Orange and pale-orange arcs show the N50 and N90 scaffold lengths (25,720,800 and 17,278,012 bp), respectively. The pale grey spiral shows the cumulative scaffold count on a log scale with white scale lines showing successive orders of magnitude. The blue and pale-blue area around the outside of the plot shows the distribution of GC, AT and N percentages in the same bins as the inner plot. A summary of complete, fragmented, duplicated and missing BUSCO genes in the lepidoptera_odb10 set is shown in the top right. An interactive version of this figure is available at https://blobtoolkit.genomehubs.org/view/ilEloNymp1_1/dataset/ilEloNymp1_1/snail.

**Figure 3.**
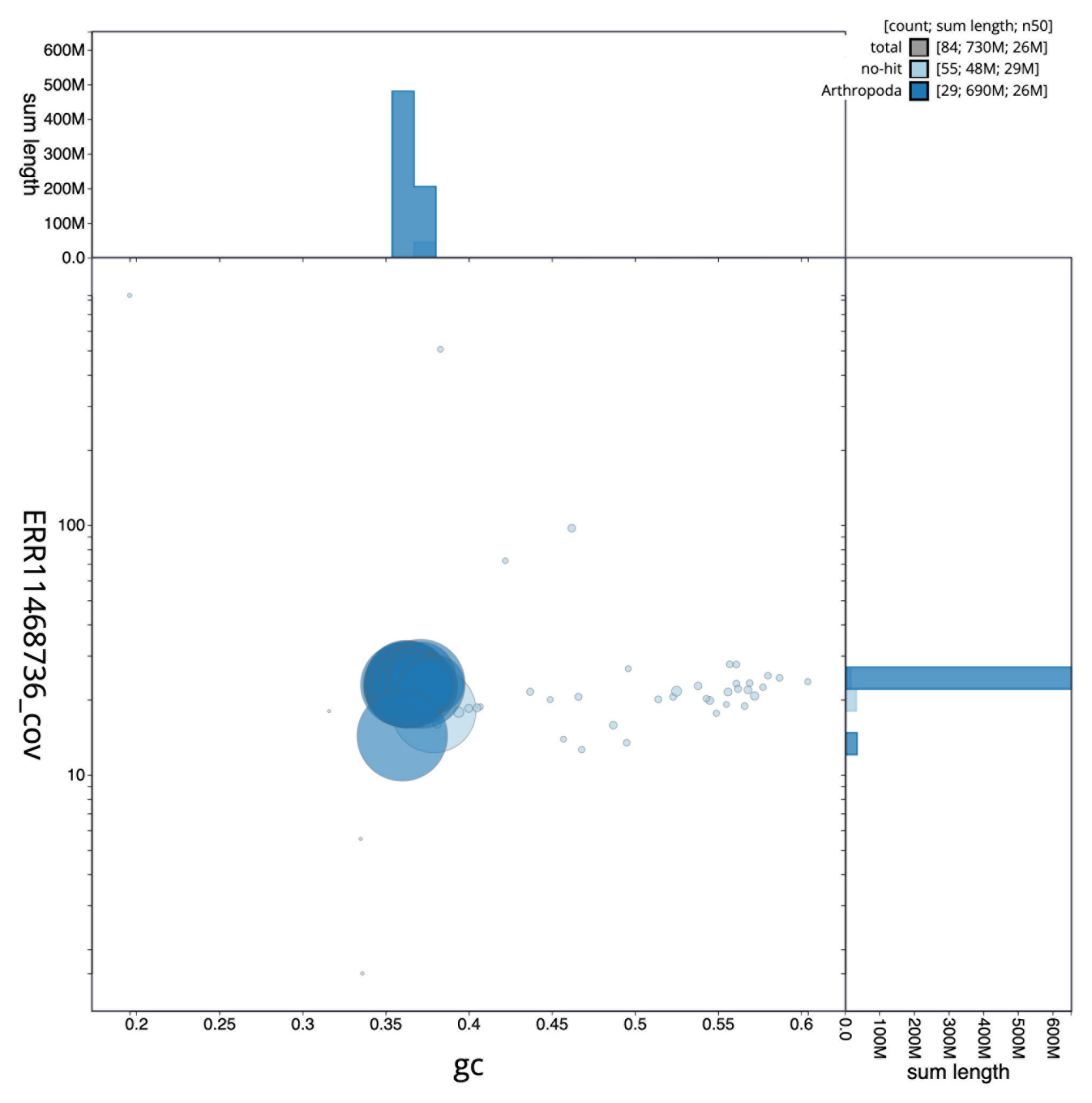
Genome assembly of *Elophila nymphaeata*, ilEloNymp1.1: BlobToolKit GC-coverage plot. Sequences are coloured by phylum. Circles are sized in proportion to sequence length. Histograms show the distribution of sequence length sum along each axis. An interactive version of this figure is available at https://blobtoolkit.genomehubs.org/view/ilEloNymp1_1/dataset/ilEloNymp1_1/blob.

**Figure 4.**
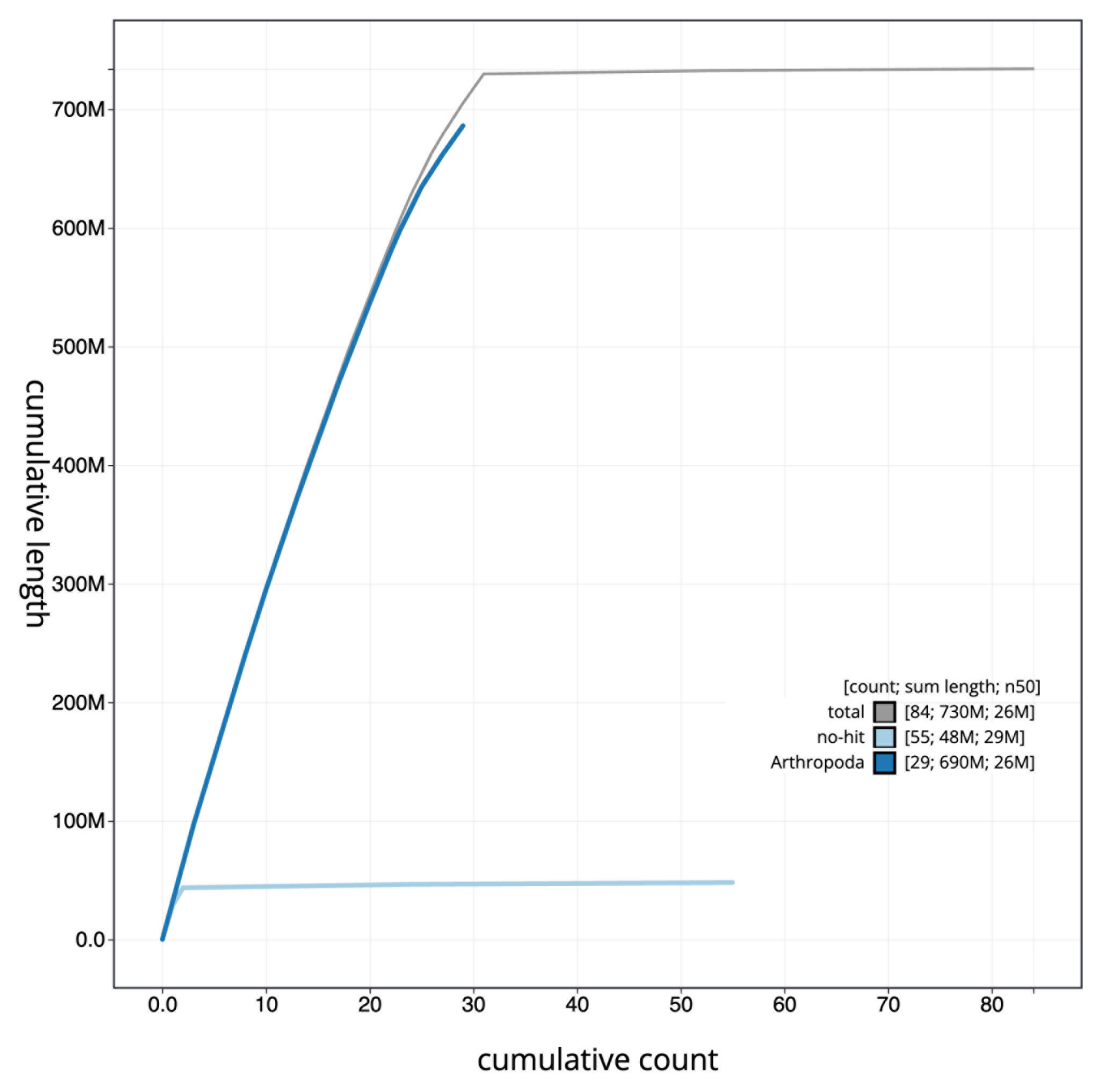
Genome assembly of *Elophila nymphaeata*, ilEloNymp1.1: BlobToolKit cumulative sequence plot. The grey line shows cumulative length for all sequences. Coloured lines show cumulative lengths of sequences assigned to each phylum using the buscogenes taxrule. An interactive version of this figure is available at https://blobtoolkit.genomehubs.org/view/ilEloNymp1_1/dataset/ilEloNymp1_1/cumulative.

**Figure 5.**
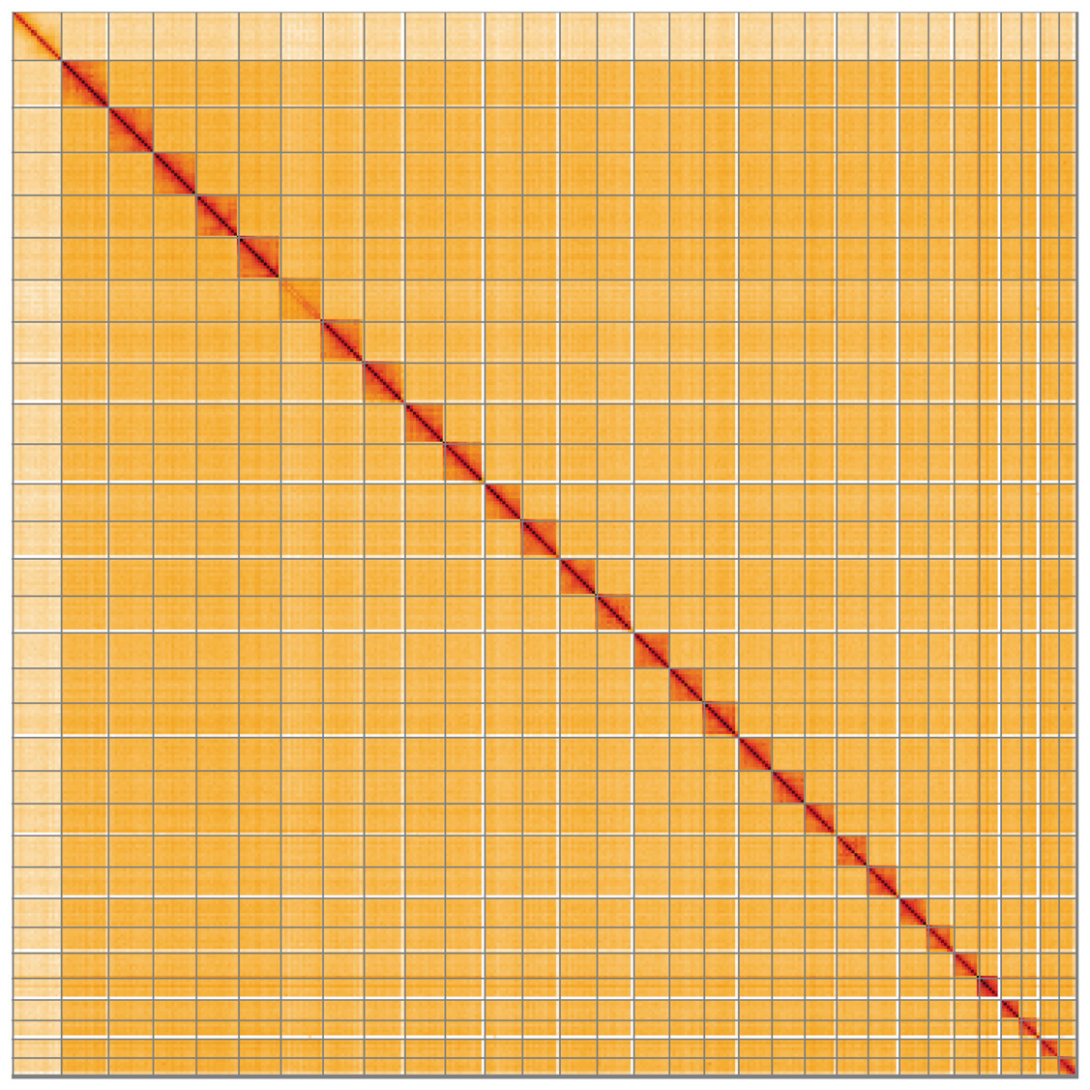
Genome assembly of *Elophila nymphaeata*, ilEloNymp1.1: Hi-C contact map of the ilEloNymp1.1 assembly, visualised using HiGlass. Chromosomes are shown in order of size from left to right and top to bottom. An interactive version of this figure may be viewed at https://genome-note-higlass.tol.sanger.ac.uk/l/?d=IgFUQX6bSK6ast2stTOrlg.

The final assembly has a total length of 734.1 Mb in 83 sequence scaffolds with a scaffold N50 of 25.7 Mb ( Table 1). The snail plot in Figure 2 provides a summary of the assembly statistics, while the distribution of assembly scaffolds on GC proportion and coverage is shown in Figure 3. The cumulative assembly plot in Figure 4 shows curves for subsets of scaffolds assigned to different phyla. Most (99.4%) of the assembly sequence was assigned to 31 chromosomal-level scaffolds, representing 29 autosomes and the Z and W sex chromosomes. The Z and W chromosomes were assigned based on half-read coverage of the PacBio reads, as well as the While not fully phased, the assembly deposited is of one haplotype. Chromosome-scale scaffolds confirmed by the Hi-C data are named in order of size ( Figure 5; Table 2). Contigs corresponding to the second haplotype have also been deposited. The mitochondrial genome was also assembled and can be found as a contig within the multifasta file of the genome submission.

**Table 1.** Genome data for *Elophila nymphaeata*, ilEloNymp1.1.

Project accession data		
Assembly identifier	ilEloNymp1.1	
Species	*Elophila nymphaeata*	
Specimen	ilEloNymp1	
NCBI taxonomy ID	753177	
BioProject	PRJEB62163	
BioSample ID	SAMEA7701303	
Isolate information	ilEloNymp1, female: whole organism (DNA sequencing) ilEloNymp2: head and thorax (Hi-C sequencing), abdomen (RNA sequencing)	
Assembly metrics *		*Benchmark*
Consensus quality (QV)	66.2	*≥ 50*
*k*-mer completeness	100.0%	*≥ 95%*
BUSCO **	C:98.7%[S:98.2%,D:0.4%],F:0.4%,M:0.9%,n:5,286	*C ≥ 95%*
Percentage of assembly mapped to chromosomes	99.4%	*≥ 95%*
Sex chromosomes	ZW	*localised homologous pairs*
Organelles	Mitochondrial genome: 15.3 kb	*complete single alleles*
Raw data accessions		
PacificBiosciences SEQUEL II	ERR11458809, ERR11458810	
Hi-C Illumina	ERR11468739	
PolyA RNA-Seq Illumina	ERR11468738	
Genome assembly		
Assembly accession	GCA_955850985.1	
*Accession of alternate haplotype*	GCA_955846215.1	
Span (Mb)	734.1	
Number of contigs	177	
Contig N50 length (Mb)	13.4	
Number of scaffolds	83	
Scaffold N50 length (Mb)	25.7	
Longest scaffold (Mb)	33.32	
Genome annotation		
Number of protein-coding genes	12,079	
Number of non-coding genes	2,124	
Number of gene transcripts	22,245	

* Assembly metric benchmarks are adapted from column VGP-2020 of “Table 1: Proposed standards and metrics for defining genome assembly quality” from Rhie *et al.* (2021). ** BUSCO scores based on the lepidoptera_odb10 BUSCO set using version 5.3.2. C = complete [S = single copy, D = duplicated], F = fragmented, M = missing, n = number of orthologues in comparison. A full set of BUSCO scores is available at https://blobtoolkit.genomehubs.org/view/ilEloNymp1_1/dataset/ilEloNymp1_1/busco.

**Table 2.** Chromosomal pseudomolecules in the genome assembly of *Elophila nymphaeata*, ilEloNymp1.

INSDC accession	Chromosome	Length (Mb)	GC%
OY039066.1	1	32.27	37.0
OY039067.1	2	30.75	36.5
OY039068.1	3	29.56	36.5
OY039069.1	4	29.1	36.5
OY039070.1	5	28.92	36.5
OY039072.1	6	28.28	36.0
OY039073.1	7	28.09	36.0
OY039074.1	8	27.48	36.0
OY039075.1	9	27.3	36.0
OY039076.1	10	25.75	36.0
OY039077.1	11	25.72	36.5
OY039078.1	12	25.57	36.5
OY039079.1	13	25.21	36.0
OY039080.1	14	24.41	36.5
OY039081.1	15	23.87	36.5
OY039082.1	16	23.66	36.5
OY039083.1	17	22.82	37.0
OY039084.1	18	22.5	36.5
OY039085.1	19	21.93	37.0
OY039086.1	20	21.6	37.0
OY039087.1	21	21.45	36.5
OY039088.1	22	19.9	37.0
OY039089.1	23	17.78	37.0
OY039090.1	24	17.28	37.0
OY039091.1	25	14.8	37.0
OY039092.1	26	14.02	37.0
OY039093.1	27	12.99	38.0
OY039094.1	28	12.81	37.5
OY039095.1	29	11.8	37.5
OY039071.1	W	28.87	38.0
OY039065.1	Z	33.32	36.0
OY039096.1	MT	0.02	20.0

The estimated Quality Value (QV) of the final assembly is 66.2 with *k*-mer completeness of 100.0%, and the assembly has a BUSCO v5.3.2 completeness of 98.7% (single = 98.2%, duplicated = 0.4%), using the lepidoptera_odb10 reference set ( *n* = 5,286).

Metadata for specimens, barcode results, spectra estimates, sequencing runs, contaminants and pre-curation assembly statistics are given at https://links.tol.sanger.ac.uk/species/753177.

## Genome annotation report

The *Elophila nymphaeata* genome assembly (GCA_955850985.1) was annotated on Ensembl Rapid Release at the European Bioinformatics Institute (EBI). The resulting annotation includes 22,245 transcribed mRNAs from 12,079 protein-coding and 2,124 non-coding genes ( Table 1; https://rapid.ensembl.org/Elophila_nymphaeata_GCA_955850985.1/Info/Index).

## Methods

### Sample acquisition and nucleic acid extraction

The specimen used for genome sequencing was a female *Elophila nymphaeata* (specimen ID Ox000536, ToLID ilEloNymp1), collected from Wytham Woods, Oxfordshire (biological vice-county Berkshire), UK (latitude 51.76, longitude –1.34) on 2020-06-25, using a light trap. The specimen was collected and identified by Douglas Boyes (University of Oxford) and preserved on dry ice.

The specimen used for Hi-C and RNA sequencing (specimen ID NHMUK014536852, ToLID ilEloNymp2) was collected from Gilbert White’s House, Selborne, UK (latitude 51.09, longitude –0.94) on 2021-06-10, using a light trap. The specimen was collected by Gavin Broad, Laura Sivess and Stephanie Holt (Natural History Museum), identified by Gavin Broad, and then dry-frozen at –80 °C.

The workflow for high molecular weight (HMW) DNA extraction at the Wellcome Sanger Institute (WSI) includes a sequence of core procedures: sample preparation; sample homogenisation, DNA extraction, fragmentation, and clean-up. In sample preparation, the ilEloNymp1 sample was weighed and dissected on dry ice ( Jay *et al.*, 2023). Tissue from the whole organism was homogenised using a PowerMasher II tissue disruptor ( Denton *et al.*, 2023a).

HMW DNA was extracted using the Automated MagAttract v1 protocol ( Sheerin *et al.*, 2023). DNA was sheared into an average fragment size of 12–20 kb in a Megaruptor 3 system with speed setting 30 ( Todorovic *et al.*, 2023). Sheared DNA was purified by solid-phase reversible immobilisation ( Strickland *et al.*, 2023): in brief, the method employs a 1.8X ratio of AMPure PB beads to sample to eliminate shorter fragments and concentrate the DNA. The concentration of the sheared and purified DNA was assessed using a Nanodrop spectrophotometer and Qubit Fluorometer and Qubit dsDNA High Sensitivity Assay kit. Fragment size distribution was evaluated by running the sample on the FemtoPulse system.

RNA was extracted from abdomen tissue of ilEloNymp2 in the Tree of Life Laboratory at the WSI using the RNA Extraction: Automated MagMax™ *mir*Vana protocol ( do Amaral *et al.*, 2023). The RNA concentration was assessed using a Nanodrop spectrophotometer and a Qubit Fluorometer using the Qubit RNA Broad-Range Assay kit. Analysis of the integrity of the RNA was done using the Agilent RNA 6000 Pico Kit and Eukaryotic Total RNA assay.

Protocols developed by the WSI Tree of Life laboratory are publicly available on protocols.io ( Denton *et al.*, 2023b).

### Sequencing

Pacific Biosciences HiFi circular consensus DNA sequencing libraries were constructed according to the manufacturers’ instructions. Poly(A) RNA-Seq libraries were constructed using the NEB Ultra II RNA Library Prep kit. DNA and RNA sequencing was performed by the Scientific Operations core at the WSI on Pacific Biosciences SEQUEL II (HiFi) and Illumina NovaSeq 6000 (RNA-Seq) instruments. Hi-C data were also generated from head and thorax tissue of ilEloNymp2 using the Arima2 kit and sequenced on the Illumina NovaSeq 6000 instrument.

### Genome assembly, curation and evaluation

Assembly was carried out with Hifiasm ( Cheng *et al.*, 2021) and haplotypic duplication was identified and removed with purge_dups ( Guan *et al.*, 2020). The assembly was then scaffolded with Hi-C data ( Rao *et al.*, 2014) using YaHS ( Zhou *et al.*, 2023). The assembly was checked for contamination and corrected as described previously ( Howe *et al.*, 2021). Manual curation was performed using HiGlass ( Kerpedjiev *et al.*, 2018) and PretextView ( Harry, 2022). The mitochondrial genome was assembled using MitoHiFi ( Uliano-Silva *et al.*, 2023), which runs MitoFinder ( Allio *et al.*, 2020) or MITOS ( Bernt *et al.*, 2013) and uses these annotations to select the final mitochondrial contig and to ensure the general quality of the sequence.

A Hi-C map for the final assembly was produced using bwa-mem2 ( Vasimuddin *et al.*, 2019) in the Cooler file format ( Abdennur & Mirny, 2020). To assess the assembly metrics, the *k*-mer completeness and QV consensus quality values were calculated in Merqury ( Rhie *et al.*, 2020). This work was done using Nextflow ( Di Tommaso *et al.*, 2017) DSL2 pipelines “sanger-tol/readmapping” ( Surana *et al.*, 2023a) and “sanger-tol/genomenote” ( Surana *et al.*, 2023b). The genome was analysed within the BlobToolKit environment ( Challis *et al.*, 2020) and BUSCO scores ( Manni *et al.*, 2021; Simão *et al.*, 2015) were calculated.

Table 3 contains a list of relevant software tool versions and sources.

**Table 3.** Software tools: versions and sources.

Software tool	Version	Source
BlobToolKit	4.2.1	https://github.com/blobtoolkit/blobtoolkit
BUSCO	5.3.2	https://gitlab.com/ezlab/busco
Hifiasm	0.16.1-r375	https://github.com/chhylp123/hifiasm
HiGlass	1.11.6	https://github.com/higlass/higlass
Merqury	MerquryFK	https://github.com/thegenemyers/MERQURY.FK
MitoHiFi	3	https://github.com/marcelauliano/MitoHiFi
PretextView	0.2	https://github.com/wtsi-hpag/PretextView
purge_dups	1.2.5	https://github.com/dfguan/purge_dups
sanger-tol/genomenote	v1.0	https://github.com/sanger-tol/genomenote
sanger-tol/readmapping	1.1.0	https://github.com/sanger-tol/readmapping/tree/1.1.0
YaHS	1.2a.2	https://github.com/c-zhou/yahs

### Genome annotation

The Ensembl Genebuild annotation system ( Aken *et al.*, 2016) was used to generate annotation for the *Elophila nymphaeata* assembly (GCA_955850985.1) in Ensembl Rapid Release at the EBI. Annotation was created primarily through alignment of transcriptomic data to the genome, with gap filling via protein-to-genome alignments of a select set of proteins from UniProt ( UniProt Consortium, 2019).

### Wellcome Sanger Institute – Legal and Governance

The materials that have contributed to this genome note have been supplied by a Darwin Tree of Life Partner. The submission of materials by a Darwin Tree of Life Partner is subject to the **‘Darwin Tree of Life Project Sampling Code of Practice’**, which can be found in full on the Darwin Tree of Life website here. By agreeing with and signing up to the Sampling Code of Practice, the Darwin Tree of Life Partner agrees they will meet the legal and ethical requirements and standards set out within this document in respect of all samples acquired for, and supplied to, the Darwin Tree of Life Project.

Further, the Wellcome Sanger Institute employs a process whereby due diligence is carried out proportionate to the nature of the materials themselves, and the circumstances under which they have been/are to be collected and provided for use. The purpose of this is to address and mitigate any potential legal and/or ethical implications of receipt and use of the materials as part of the research project, and to ensure that in doing so we align with best practice wherever possible. The overarching areas of consideration are:

•     Ethical review of provenance and sourcing of the material

•     Legality of collection, transfer and use (national and international) 

Each transfer of samples is further undertaken according to a Research Collaboration Agreement or Material Transfer Agreement entered into by the Darwin Tree of Life Partner, Genome Research Limited (operating as the Wellcome Sanger Institute), and in some circumstances other Darwin Tree of Life collaborators.

## Data Availability

European Nucleotide Archive: *Elophila nymphaeata* (brown china-mark). Accession number PRJEB62163; https://identifiers.org/ena.embl/PRJEB62163 ( Wellcome Sanger Institute, 2023). The genome sequence is released openly for reuse. The *Elophila nymphaeata* genome sequencing initiative is part of the Darwin Tree of Life (DToL) project. All raw sequence data and the assembly have been deposited in INSDC databases. Raw data and assembly accession identifiers are reported in Table 1.

## Author information

Members of the University of Oxford and Wytham Woods Genome Acquisition Lab are listed here: https://doi.org/10.5281/zenodo.7125292.

Members of the Natural History Museum Genome Acquisition Lab are listed here: https://doi.org/10.5281/zenodo.7139035.

Members of the Darwin Tree of Life Barcoding collective are listed here: https://doi.org/10.5281/zenodo.4893703.

Members of the Wellcome Sanger Institute Tree of Life Management, Samples and Laboratory team are listed here: https://doi.org/10.5281/zenodo.10066175.

Members of Wellcome Sanger Institute Scientific Operations: Sequencing Operations are listed here: https://doi.org/10.5281/zenodo.10043364.

Members of the Wellcome Sanger Institute Tree of Life Core Informatics team are listed here: https://doi.org/10.5281/zenodo.10066637.

Members of the Tree of Life Core Informatics collective are listed here: https://doi.org/10.5281/zenodo.5013541.

Members of the Darwin Tree of Life Consortium are listed here: https://doi.org/10.5281/zenodo.4783558.
