# Supplementary material for: The genome sequence of the Brown China-mark moth, Elophila nymphaeata (Linnaeus, 1758)
Source: Wellcome Open Res. 2024 Mar 19;9:155. [Version 1] doi: 10.12688/wellcomeopenres.21118.1 (PMC12447070; doi:10.12688/wellcomeopenres.21118.1)
Supplement: Peer review history

# Reviewer report: Reviewer response for version 1

**DOI:** 10.21956/wellcomeopenres.23360.r131485

**Published:** 2025-09-18

**Authors:** Sara E Mousa, Gehad N Aboulnasr

**Affiliations:** 1 Zoology and Entomology Department, Faculty of Science, Assiut University, Asyut, Assiut Governorate, Egypt; 2 Plant Protection, Faculty of Agriculture, Assiut University, Asyut, Assiut Governorate, Egypt

The primary importance of sequencing the Elophila nymphaeata genome lies in its unusual aquatic lifestyle. While the vast majority of moths and butterflies (Lepidoptera) have terrestrial larvae, the Brown China-mark moth is one of the few species whose larvae are fully aquatic, living underwater and feeding on submerged plants. This aquatic lifestyle is believed to have evolved secondarily, meaning its ancestors were terrestrial.

 Therefore, having a high-quality reference genome for this species is highly valuable for scientific research. It provides a crucial resource for comparative genomics, allowing scientists to study the specific genetic adaptations that enabled insects to transition from a terrestrial to an aquatic environment. This research will also contribute to the broader understanding of molecular evolution within the Lepidoptera order.

Are sufficient details of methods and materials provided to allow replication by others?

Yes

Is the rationale for creating the dataset(s) clearly described?

Yes

Are the datasets clearly presented in a useable and accessible format?

Yes

Are the protocols appropriate and is the work technically sound?

Yes

Reviewer Expertise:

Plant Protection, Economic Entomology, Biological Control, Molecular Genetics, Ecological Studies

We confirm that we have read this submission and believe that we have an appropriate level of expertise to confirm that it is of an acceptable scientific standard.

# Reviewer report: Reviewer response for version 1

**DOI:** 10.21956/wellcomeopenres.23360.r87658

**Published:** 2024-08-06

**Authors:** Jacqueline Heckenhauer

**Affiliations:** 1 Senckenberg Research Institute, Frankfurt, Germany

The submission outlines sequencing efforts, genome assembly, and annotation of a previously unsequenced Lepidoptera species. This study has generated high-quality data that is openly available. It thus is a valuable addition to the records of genome descriptions from Lepidoptera with an aquatic larval stage and might be used to study adaptations to aquatic environments.

Are sufficient details of methods and materials provided to allow replication by others?

Yes

Is the rationale for creating the dataset(s) clearly described?

Yes

Are the datasets clearly presented in a useable and accessible format?

Yes

Are the protocols appropriate and is the work technically sound?

Yes

Reviewer Expertise:

biodiversity genomics, evolution, aquatic insects

I confirm that I have read this submission and believe that I have an appropriate level of expertise to confirm that it is of an acceptable scientific standard.
